# Efficient and easy gene expression and genetic variation data analysis and visualization using exvar

**DOI:** 10.1038/s41598-025-93067-5

**Published:** 2025-04-10

**Authors:** Hiba Ben Aribi, Imraan Dixon, Najla Abassi, Olaitan I. Awe

**Affiliations:** 1 Faculty of Sciences of Tunis, University of Tunis El Manar, Tunis, Tunisia; 2 Faculty of Health Sciences, University of Cape Town, Cape Town, South Africa; 3 Higher Institute of Biotechnology Sidi Thabet, Manouba University, Manouba, Tunisia; 4 Department of Computer Science, University of Ibadan, Ibadan, Oyo State Nigeria; 5 African Society for Bioinformatics and Computational Biology, Cape Town, South Africa

**Keywords:** Exvar, Gene expression, Variants calling, CNVs, SNPs, Indels, R package, Computational biology and bioinformatics, Genetics

## Abstract

RNA sequencing data manipulation workflows are complex and require various skills and tools. This creates the need for user-friendly and integrated genomic data analysis and visualization tools. We developed a novel R package using multiple Cran and Bioconductor packages to perform gene expression analysis and genetic variant calling from RNA sequencing data. Multiple public datasets were analyzed using the developed package to validate the pipeline for all the supported species. The developed R package, named “exvar”, includes multiple data analysis functions and three data visualization shiny apps integrated as functions. Also, it could be used to analyze several species’ data. The exvar package is available in the project’s GitHub repository (https://github.com/omicscodeathon/exvar).

## Introduction

High-throughput sequencing technologies play a crucial role in biological and medical research. Gene expression profiling is crucial to understanding the molecular mechanisms of pathologies since the differentially expressed genes (DEGs) represent potential biomarkers and therapeutic targets^1^. This pursuit is exemplified by recent studies like Nyamari et al.^2^ on the ACE2 receptor and Chikwambi et al.^3^ on prostate cancer, highlighting the potential of gene expression data in biomarker discovery.

Genomic variants represent major factors in pathology occurrence, susceptibility, and drug response^4^. Multiple types of genome variants exist, including Single Nucleotide Polymorphism (SNPs), Insertions or deletions (Indels), and copy number variations (CNVs). Ninety percent of human DNA is SNPs^5^. SNPs can be an insertion, deletion, or substitution in comparison to the reference genome^6^. Nucleotide insertions or deletions can affect both long segments of a chromosome or simply one single nucleotide, the latter being a particular form of indels^7^. Indels impact the number of nucleotides in a DNA sequence and influence gene function, expression, and coded protein^8–11^. Copy number variations can cause a dosage imbalance of one or more genes^12^. Multiple benign CNVs exist in the human genome without any association with pathologies^13^, however, recent large population studies have associated numerous CNVs with various diseases^14^.

RNA sequencing data manipulation workflows, from raw Fastq files to biologically significant information, are complex and require various skills and tools. Several R packages exist for gene expression and genetic variation analysis, each with specific strengths and limitations. For example, geneHummus^15^ focuses on gene family analysis, while limma (Ritchie et al*.,* 2015) is widely used for differential expression analysis. Other tools focus on educating users about sequencing data analysis, such as SeqAcademy, an educational pipeline developed by Ather et al.^16^ that exemplifies the need for user-friendly resources to equip researchers with the necessary skills for RNA-seq and ChIP-seq analysis.

This study aimed to develop a novel R package for genetic data analysis and visualization, to be used by clinicians and biologists with basic programming skills. The exvar package complements existing tools by offering a user-friendly interface for gene expression and genetic variation analysis, making it accessible to a wider range of users.

## Methods

The “exvar” package is a novel package developed using the “devtools” in R^17,18^ and documented using the Roxygen2 package^17,18^. The package includes six data analysis functions, three data visualization functions, and a function dependencies installation.

### Data preprocessing function

The **processfastq()** function requires fastq files as input. The quality control is performed using the rfastp package^19^ generating a JSON report file and a quality summary in CSV format. Reads longer than 200 bases are trimmed due to limitations associated with the reference genome creation step. The Fatsq files are aligned to a reference genome created using the gmapR package^20^. The resulting outputs are indexed BAM files.

### Gene expression analysis function

The **expression()** function seeks the directories created by the processfastq() function for BAM files. These BAM files are labeled accordingly and gene counts are extracted using the GenomicAlignments package^21^ and used by the DESeq2 packages^22^ to create a DESeq object with which differential expression analysis can be conducted. The advancements in RNA sequencing have enabled differential gene expression analysis from transcriptomic datasets^23–26^. Gene symbols are attached to gene IDs obtained from a TxDb object for gene counting. A CSV file is created for the differential expression analysis.

The **counts()** function works the same as the expression() function, but instead of performing the differential expression analysis, it stops at extracting the gene counts and outputting a CSV file containing the count data.

### Variant calling functions

The **callsnp()** function uses the VariantTools package^27^ with the reference genome created with gmapR acting as a reference. Variants represent the differences between a reference sequence and other sequences^28–30^. It takes as input a BAM file and creates a VCF file indicating variant information. The resulting VCF is injected with corresponding dbSNP IDs stored within a SNPlocs object^31^ using the VariantAnnotation package^32^.

The **callindel()** function calls the indels similarly to SNPs using the VariantTools package^27^. While the function in VariantTools defaults to exclude indels, changing a boolean parameter allows indel calling. Thus, like callsnp(), callindel() receives a BAM file as an input and outputs a VCF containing only indels using the VariantAnnotation package^32^.

The **callcnv()** function creates a bed file from a TxDb object using the tracklayer package^33^. This informs the regions in which read counts are piled up using the panelcn.mops package^34^. Copy number variation is estimated from a BAM file, requiring at least one BAM file from a control sample in addition to the sample in which the copy number variation is to be estimated. The function outputs a CSV file containing the copy number states of genomic regions.

### Data visualization functions

The data visualization functions are shiny applications. The applications’ interfaces were developed using the shiny R package^35^. Other packages are used for data management and the applications’ dashboard creation (The complete list of these packages and their citation are provided in the supplementary material document 2).

**Vizexp()** function requires the gene counts data, as a CSV file, and a metadata file, as inputs, for the expression data visualization. The expression analysis is performed using the DESeq2 packages^22^ and the differentially expressed genes are identified based on the *p*-value (or adjusted *p*-value) and the logFC value, defined by the user. The data is visualized in an MA plot, a PCA plot, and a volcano plot created using the ggplot2 package^36^. Gene ontology (GO) enrichment analysis is performed using the AnnotationDbi^37^ and the ClusterProfiler^38^ packages. The statistically significant ontologies are represented in barplots, dotplots, and cnet plots using the Enrichplot package^39^.

For its inputs, the **vizsnp()** function requires the path to the directory containing the variants’ data VCF files. The files are expected to be in two folders, one containing the control group files and another containing the patient group files. The common SNPs between all samples for each group are identified, then the two groups’ data are compared. The SNPs with different nucleotides between the patient and control are identified. The SNP types and the transitions/transversions are identified and represented in barplots, using the ggplot2 package^36^. The function also detects SNPs, in comparison with the reference genome that exists in either the patient or control group samples only. DESEq2 or edgeR does not have the vizualisation part. VCFtools does not have a visualization component. For exvar, the user does not have to go through a variant calling pipeline step by step. Other tools like PRESM do not have a vizualisation component while exvar does have visualization components.

The **vizcnv()** function requires the variants’ data CSV file as input. The target chromosome number is defined by the user. The CNVRanger package^40^ is used to read the CNVs data, and the low-density areas are trimmed using the CNVRuler approach^41^. The recurrent regions are identified using the GISTIC method^42^, and the CNVs that exceed the significant threshold, defined by the user, are filtered. The recurrent CNV region landscape is illustrated using the CNVRanger package^40^. The target gene type is also required as input (protein coding, pseudogene, rRNA, miRNA, miscRNA, snRNA, or snoRNA). The target genes’ location is obtained from the Ensemble database using the AnnotationHub package^43^. The GenomicRanges package is used to find the overlaps of CNVs with functional genomic regions. The original CNV calls on overlapping genomic features are illustrated in an oncoPrint plot. The Overlap permuted test is performed using the regioneR^44^ and illustrated.

### Supported species

The primary objective in developing this package was to provide support for eight species, namely *Homo sapiens, Mus musculus, Arabidopsis thaliana, Drosophila melanogaster, Danio rerio, Rattus norvegicus, Caenorhabditis elegans, *and *Saccharomyces cerevisiae*. The species-specific packages used for each function and species are documented in the supplementary material document 2. However, due to the limitation of the absence of dedicated species-specific open-source R packages for certain organisms within the current framework, not all functions support all the species.

### Pipeline validation

Pipeline validation was performed on publicly available datasets on SRA database, including SRP074425 (Wang et al., 2016) and SRR24633043 (Choi et al*.,* 2023) for Homo Sapiens, SRP428998 for Mus Musculus (Foreman et al*.,* 2023), SRP091721 for Rattus norvegicus (Krebber et al*.,* 2020), SRP310413 for Arabidopsis Thaliana (Fan et al*.,* 2021), SRP310413 for Drosophila Melanogaster^38^, SRP254020 for Danio rerio (Ai et al*.,* 2022), SRP427377 for Saccharomyces Cerevisiae (Sosa Ponce et al*.,* 2023), and SRP434916 (Zheng, 2023) for Caenorhabditis elegans. All functions were validated using the analyzed data, except the vizcnv() function which was validated using simulation data for species other than Homo Sapiens.

## Results

The exvar package provides functions for Fastq files preprocessing, and gene expression and genetic variation (SNPs, Indels, and CNVs) data analysis and visualization. It could be used to analyze data from eight species including *Homo Sapiens*, *Mus Musculus, Arabidopsis Thaliana, Drosophila Melanogaster, Danio rerio, Rattus norvegicus, Caenorhabditis elegans,* and *Saccharomyces Cerevisiae.* The exvar package is publicly available on the project GitHub repository and can be installed using this command:

devtools::install_github(“omicscodeathon/exvar/Package”)

We have packaged exvar as a standalone package of software that contains everything needed to run it. This contains the code, software dependencies and configuration files. A docker container is available for exvar and so, the URL for the docker container is:

docker://imraandixon/exvar

The container can be pulled from the Docker Hub using the following command :

docker pull imraandixon/exvar

The package consists of 6 data analysis functions (processfastq(); counts(); expression(); callsnp(); callcnv(); and callindel()), 3 data visualization functions (vizexp(), vizsnp(), and vizcnv()), and a requirement() function is also provided to install all the dependencies. The list of functions and their roles are represented in Table 1. A detailed step-by-step guide to using each function is provided in the supplementary material document 1.

**Table 1:** exvar package functions information.

Function	Role	Input	Output
requirement()	Install required packages	–	–
processfastq()	Preprocess fastq files	Fastq files	BAM files
counts()	Gene count analysis	BAM files	CSV file
expression()	Identify DEGs	BAM files	CSV file
callsnp()	SNP calling	BAM files	VCF files
callcnv()	CNV calling	BAM files	CSV file
callindel()	Indel calling	BAM files	VCF files
vizexp()	Analyze and visualize gene expression data	CSV file	Interactive interface
vizsnp()	Analyze and visualize SNP data	VCF files	Interactive interface
vizcnv()	Analyze and visualize CNV data	CSV file	Interactive interface

The **requirement()** function will install all the required packages for the package functioning, depending on the target species and the machine operating system. The **processfastq()** function produces a JSON report of the fastq quality control step. This report details the number of reads filtered, their length, and the GC content of those reads. It also details the read quality and kmer counts before and after filtering. The function filters the reads longer than 200 and then aligns the processed fastq files to a reference genome of choice. The result is an indexed BAM file created in the same directory as the fastq file.

The **expression()** function parses the folder set in the parameters as part of the dir parameter. First, it assumes that all BAM files are found in separate folders as would be the case if processfastq() were to align fastq files. Second, it assumes that the folders containing each sample’s BAM file would be in separate folders. It creates CSV files containing the differential expression analysis of each group with each other, with each group being named after the folder they are found in. The **counts()** function works similarly to the expression() function but outputs CSV files containing gene expression counts of BAM files.

The **callsnp()** and **callindel()** functions both create VCF files containing SNPs and Indels variants respectively. Both detail the allelic depth and read depth of each variant. The **callcnv()** function creates a CSV file of copy number counts that can be read by the **vizcnv()** function.

The **vizexp()** function first visualizes the count data before and after the log transformation, for quality control purposes, (Supplementary material document 2, Fig. 1). Second, the *p*-value (or adjusted *p*-value) and the LogFC value are requested to define the differentially expressed genes. The expression analysis result is visualized in a volcano plot, MA plot, and PCA plot (Supplementary material document 2, Fig. 2). Third, ontology analysis is performed. The target gene group (all DEGs, down-regulated genes, or up-regulated genes), the top statistically significant ontologies number to include in the plot, the ontology type (biological processes, cellular components, or molecular function), and the plot type (dotplot, barplot, or cnetplot) are defined by the user (Supplementary material document 2, Fig. 3).

The **vizsnp()** function parses the folder set in the parameters as part of the dir parameter. First, it assumes that all VCF files are found in separate folders as would be the case if callsnp() were to generate the files. Second, it assumes that the folders are named “control” and “patient” containing the VCF files of the control and patient group samples respectively. Third, the function identifies the SNPs that are different between the two groups. The distribution of SNPs is visualized in two barplots (Supplementary material document 2, Fig. 4.1). It also identifies the SNPs existing in each group in comparison with the reference genome (Supplementary material document 2, Fig. 4.2).

The **vizcnv()** function first requests the target chromosome and the significant *p*-value to define the recurrent CNVs, then the recurrent CNV regions are identified and represented (Supplementary material document 2, Fig. 5). Second, the CNVs-genes overlapping analysis result is illustrated in an oncoPrint plot (Supplementary material document 2, Fig. 6). Third, the overlap permutation test result is illustrated (Supplementary material document 2, Fig. 7).

All the plots and csv files generated by the vizexp(), vizsnp(), and vizcnv() functions can be downloaded from the applications.

The package exvar reduces the need for user intervention between the analysis steps. For example:

- Creating a full pipeline using compatible packages. We did the research to select peer reviewed tools (reviewed by journals or bioconductor), to reduce the effort needed by new bioinformaticians.
- Performing annotation automatically without need of doing it independently
- Creating the correct file format of the output to be compatible with the next step or function
- Creating interactive user interfaces when possible to reduce the code based intervention
- Condensing full pipelines into a unique function

Overall, exvar might take the same time or memory to execute a pipeline but it only requires the user to run at maximum 10 lines of code (number of exvar functions) in order to perform the most common genetic data analysis and visualizations.

### Limitations

The package is developed using established open-source Cran and Bioconductor packages, however, not all functions seamlessly apply to every species due to the inherent biological variations among organisms. This limitation arises from the absence of dedicated species-specific packages for certain organisms within the current framework.

The exvar package presently incorporates functionality tailored to eight distinct species, namely *Homo sapiens, Mus musculus, Arabidopsis thaliana, Drosophila melanogaster, Danio rerio, Rattus norvegicus, Caenorhabditis elegans, *and *Saccharomyces cerevisiae*. However, it is important to note that certain functions may not be universally available for all species, mainly the callcnv() and vizcnv()functions. Table 2 specifies the species supported by each function.

**Table 2:** List of species supported by each function of the exvar package.

Function/ Species	processfastq	counts	expression	callsnp	callcnv	callindel	vizexp	vizsnp	vizcnv
*Homo sapiens*	✓	✓	✓	✓	✓	✓	✓	✓	✓
*Mus Musculus*	✓	✓	✓	✓	✗	✓	✓	✓	✓
*Arabidopsis Thaliana*	✓	✓	✓	✓	✗	✓	✓	✓	✗
*Drosophila Melanogaster*	✓	✓	✓	✓	✗	✓	✓	✓	✓
*Danio rerio*	✓	✓	✓	✓	✗	✓	✓	✓	✓
*Rattus norvegicus*	✓	✓	✓	✓	✗	✓	✓	✓	✓
*Caenorhabditis elegans*	✓	✓	✓	✓	✗	✓	✓	✓	✗
*Saccharomyces Cerevisiae*	✓	✓	✓	✓	✗	✓	✓	✓	✓

Additionally, due to the dependencies and specific operating system requirements, the processfastq(), counts(), expression(), callsnp(), callcnv(), and callindel() functions require a Linux operating system. However, the visualization functions vizexp(), vizsnp(), and vizcnv() are platform-independent.

## Discussion

Numerous R packages exist for RNA sequencing data analysis. However, multiple functions from different packages are required to perform gene expression or variant calling analysis and data visualization. The exvar package integrates all the required pipelines in ten functions to simplify the tasks for biologists and clinicians with basic programming skills. The way exvar makes the pipeline faster or efficient or simpler is by reducing the need for user intervention between the analysis steps. To highlight the novelty of the package we could compare it to the most commonly used variant calling tool GATK^45^, which was designed primarily for Next Generation Sequencing (NGS) variant analysis. Unlike GATK, exvar integrates differential expression analysis tools, an integration absent in GATK during the exvar development period, in addition to the genomic variant analysis. Also, GATK4 does not include any data visualization features while exvar comes built-in with visualization functions that further reduce the need for advanced programming skills. The package exvar is not specific to any disease, such as the PRESM tool^46^ which is used for detecting cancer-related variations. Thus exvar could be utilised to analyze data from diverse types of organisms or diseases. The vizexp(), vizsnp(), and vizcnv() functions are interactive shiny apps, that integrate multiple analyses with settings customization options, and show biologist-friendly web interfaces. Additionally, the exvar package could be used to analyze data from multiple species, while most available tools are specific to the *Homo sapiens* species.

## Conclusions

The package exvar is a novel R package, designed with a clear aim to provide a unique combination of gene expression and genomic variant analysis and visualization features for biologists with limited programming skills. The user can perform multiple analyses and visualize biological data without needing any third-party program. However, it is important to acknowledge certain limitations inherent in its design. The package, developed using established open-source Cran and Bioconductor packages, encounters challenges in applying certain functions across all species due to inherent biological variations and the absence of dedicated species-specific packages within the current framework. Despite these limitations, exvar stands as an open-source tool, publicly available on the project’s GitHub repository, offering a promising platform for collaborative contributions and ongoing development to address these challenges and enhance its functionality for a broader range of biological applications.

## Supplementary Information

Supplementary Information.

## Data Availability

The datasets generated and/or analysed during the current study are available in the GitHub repository, https://github.com/omicscodeathon/exvar.

## Declarations

### Reporting issues

To report an issue please use the issues page (https://github.com/omicscodeathon/exvar/issues). Please check existing issues before submitting a new one.
