# Supplementary material for: Efficient and easy gene expression and genetic variation data analysis and visualization using exvar
Source: Sci Rep. 2025 Apr 10;15:12264. doi: 10.1038/s41598-025-93067-5 (PMC11985497; doi:10.1038/s41598-025-93067-5)
Supplement: Supplementary file 1 — Supplementary Information. [file 41598_2025_93067_MOESM1_ESM.docx]

**Supplementary material for**

**Efficient and Easy Gene Expression and Genetic Variation Data Analysis and Visualization using exvar**

Hiba Ben Aribi^1^, Imraan Dixon^2^ , Najla Abassi^3^, and Olaitan I. Awe^4,5^

^1^ Faculty of Sciences of Tunis, University of Tunis El Manar, Tunis, Tunisia

^2^ Faculty of Health Sciences, University of Cape Town, Cape Town, South Africa

^3^ Higher Institute of Biotechnology Sidi Thabet, Manouba University, Tunisia

^4^ Department of Computer Science, University of Ibadan, Ibadan, Oyo State, Nigeria

^5^ African Society for Bioinformatics and Computational Biology, Cape Town, South Africa

**Abstract**

RNA sequencing data manipulation workflows are complex and require various skills and tools. This creates the need for user-friendly and integrated genomic data analysis and visualization tools.

We developed a novel R package using multiple Cran and Bioconductor packages to perform gene expression analysis and genetic variant calling from RNA sequencing data. Multiple public datasets were analyzed using the developed package to validate the pipeline for all the supported species.

The developed R package, named “exvar”, includes multiple data analysis functions and three data visualization shiny apps integrated as functions. Also, it could be used to analyze several species’ data.

The exvar package is available in the project’s GitHub repository (https://github.com/omicscodeathon/exvar).

**Keywords:** exvar, Gene expression, Variants calling, CNVs, SNPs, Indels, R package.

# **Abbreviations**

DEGs: Differentially Expressed Genes

CNV: Copy Number Variation

SNP: Single Nucleotide Polymorphism

INDEL: Insertion Deletion

CSV: Comma Separated Values

VCF: Variant Call Format

BAM: Binary Alignment Map

**The Package exvar Functions Documentation**

The package consists of six data analysis functions (processfastq(); counts(); expression(); callsnp(); callcnv(); and callindel()), three data visualization functions (vizexp(), vizsnp(), and vizcnv()), and a function for dependencies installation (requirements()).

1. **requirements() function**

**Description**

This function will install and call the required packages according to the target species.

**Usage**

requirements()

**Arguments**

NA

1. **processfastq() function**

**Description:** This function takes in FASTQ files and performs quality control before aligning to a reference genome. It assumes paired-end samples are of the same file name with an underscore (_) and a number to signify different reads of the same sample. Each sample's outputs will be stored in a separate directory.

**Usage**

processfastq( file = list_files_with_exts(dir = dir, exts = "fastq"), dir = getwd(), genome, genomedir, paired = FALSE, threads = 4L, molecule = "RNA" )

**Arguments**

| file | A list of paths to FASTQ files. If no paths are entered, it defaults to all fastq files in dir. |
| --- | --- |
| dir | Output directory. |
| genome | A BSgenome object, GmapGenome object, or a character string indicating the genome name eg. "hg19". |
| genomedir | A directory containing the reference genome. Otherwise, it is the parent directory of the reference genome where the genome is a character string or BSgenome object. |
| paired | Indicates whether the samples are from paired-end or single-end reads. |
| threads | The number of cores to use in the process. |
| molecule | A character string indicating either DNA or RNA samples. |

**Value**

A list of file paths to created BAM files

1. **counts() function**

**Description**

This function counts reads of gene regions between sample groups. It assumes that sample BAM files are ordered in a directory structure such as "group/sample/" as processfastq() would order it. It outputs a CSV file showing gene counts. Works similarly to expression(), but outputs count data instead of differential expression data.

**Usage**

counts( dir = getwd(), groups, TxDb, orgDb, outputdir = getwd(), threads = 4L, paired = FALSE )

**Arguments**

| dir | The parent directory of the sample groups. |
| --- | --- |
| groups | Folder names of the sample groups. The default is all folders in dir. |
| TxDb | A TxDb object upon which regions of the genome are counted. |
| orgDb | An orgDb object for annotating the CSV with gene symbols and Ensembl IDs. |
| outputdir | Output directory of CSV file. |
| threads | Number of cores to use. |
| paired | Indicates whether the samples are from paired-end reads. |

**Value**

A data frame containing gene counts.

1. **expression() function**

**Description**

This function analyzes differentially expressed genes between sample groups. It assumes that sample BAM files are ordered in a directory structure such as "group/sample/" as processfastq() would order it. There should be more than one sample per group or else differential expression analysis won't work. It outputs a CSV file showing differential expression (ordered by p-value). It works similarly to counts(), but then further analyzes those counts to obtain differential expression data.

**Usage**

expression( dir = getwd(), groups, TxDb, orgDb, outputdir = getwd(), threads = 4L, paired = FALSE )

**Arguments**

| dir | The parent directory of the sample groups. |
| --- | --- |
| groups | Folder names of the sample groups. The default is all folders in dir. |
| TxDb | A TxDb object upon which regions of the genome are counted. |
| orgDb | An orgDb object for annotating the CSV with gene symbols and Ensembl IDs. |
| outputdir | Output directory of CSV file. |
|  |  |
| threads | Number of cores to use. |
| paired | Indicates whether the samples are from paired-end reads. |

**Value**

A data frame list containing all of the differential expression comparisons.

1. **callsnp() function**

**Description**

This function calls single nucleotide polymorphism variants from BAM files. The results are formatted into a VCF file and the ID column is populated with dbSNP IDs.

**Usage**

callsnp(bam, genome, genomedir, SNPlocs, threads = 4L, outputdir = getwd())

**Arguments**

| bam | A list of paths to BAM files |
| --- | --- |
| genome | A BSgenome object, a GmapGenome object, or a character string indicating the reference genome eg. "hg19" |
| genomedir | The directory containing the reference genome or, if genome is a character string, the parent directory of the reference genome directory. |
| SNPlocs | An SNPlocs object containing dbSNP IDs. |
| threads | The number of cores to use. |
| outputdir | The output directory for the VCF file. |

**Value**

A list of file paths to the VCF files.

1. **callcnv() function**

**Description**

This function calls copy number variants from sample BAM files compared to control BAM files. It assumes that BAM files are stored in separate folders as is created by processfastq(). This function requires that control BAM files are provided. Once complete, it creates a CSV file containing copy number information.

**Usage**

callcnv( controldir, control = NULL, experimentdir, experiment = NULL, bed, outputdir = getwd() )

**Arguments**

| controldir | The parent directory of the sample directories. |
| --- | --- |
| control | The names of the folders in which control BAM files are. If NULL, all folders in controldir will be checked for BAM files. |
| experimentdir | The parent directory of the sample on which to investigate copy numbers. |
| experiment | The names of the folders in which sample BAM files are. If NULL, all folders in experimentdir will be checked for BAM files. |
| bed | A character string indicating BED file path or a TxDb object from which to extract a BED file. |
| outputdir | The directory in which to place the copy number call. |

**Value**

A data frame containing copy number calls.

1. **callindel() function**

**Description**

This function calls indel variants from BAM files. The results are formatted into a VCF file and the ID column is populated with dbSNP IDs.

**Usage**

callindel(bam, genome, genomedir, SNPlocs, threads, outputdir = getwd())

**Arguments**

| bam | A list of paths to BAM files |
| --- | --- |
| genome | A BSgenome object, a GmapGenome object, or a character string indicating the reference genome eg. "hg19" |
| genomedir | The directory containing the reference genome or, if genome is a character string, the parent directory of the reference genome directory. |
| SNPlocs | An SNPlocs object containing dbSNP IDs. For indels, this may be an XtraSNPlocs object. |
| threads | The number of cores to use. Cores should equal a factor of reference genome sequence levels ie. chromosome contigs should be equally divisible between cores. |
| outputdir | The output directory for the VCF file. |

**Value**

A list of file paths to the VCF files.

1. **vizexp() function**

**Description**

This function visualizes expression data from a CSV file.

**Usage**

vizexp(genecount, metadata)

**Arguments**

| genecount | The count data csv file |
| --- | --- |
| metadata | The metadata excel file. |

1. **vizsnp()**

**Description**

This function visualizes SNPs data from a VCF file.

**Usage**

vizsnp(dir = getwd())

**Arguments**

dir The parent directory of the files (expected to include two folders named “control” and “patient”)

1. **vizcnv()**

**Description**

This function visualizes CNVs data from a VCF file.

**Usage**

vizcnv( cnvdata)

**Arguments**

cnvdata The path to variant data VCF file.
